# Developing consensus-based policy solutions for medicines adherence for Europe: a delphi study

**DOI:** 10.1186/1472-6963-12-425

**Published:** 2012-11-23

**Authors:** Wendy Clyne, Simon White, Sarah McLachlan

**Affiliations:** 1 School of Pharmacy, Keele University, Staffordshire, ST5 5BG, UK

**Keywords:** Policy, Consensus, Delphi technique, Medication adherence

## Abstract

**Background:**

Non-adherence to prescribed medication is a pervasive problem that can incur serious effects on patients’ health outcomes and well-being, and the availability of resources in healthcare systems. This study aimed to develop practical consensus-based policy solutions to address medicines non-adherence for Europe.

**Methods:**

A four-round Delphi study was conducted. The Delphi Expert Panel comprised 50 participants from 14 countries and was representative of: patient/carers organisations; healthcare providers and professionals; commissioners and policy makers; academics; and industry representatives. Participants engaged in the study remotely, anonymously and electronically. Participants were invited to respond to open questions about the causes, consequences and solutions to medicines non-adherence. Subsequent rounds refined responses, and sought ratings of the relative importance, and operational and political feasibility of each potential solution to medicines non-adherence. Feedback of individual and group responses was provided to participants after each round. Members of the Delphi Expert Panel and members of the research group participated in a consensus meeting upon completion of the Delphi study to discuss and further refine the proposed policy solutions.

**Results:**

43 separate policy solutions to medication non-adherence were agreed by the Panel. 25 policy solutions were prioritised based on composite scores for importance, and operational and political feasibility. Prioritised policy solutions focused on interventions for patients, training for healthcare professionals, and actions to support partnership between patients and healthcare professionals. Few solutions concerned actions by governments, healthcare commissioners, or interventions at the system level.

**Conclusions:**

Consensus about practical actions necessary to address non-adherence to medicines has been developed for Europe. These actions are also applicable to other regions. Prioritised policy solutions for medicines non-adherence offer a benefit to policymakers and healthcare providers seeking to address this multifaceted, complex problem.

## Background

Many patients do not take prescribed medication as advised [1]: the World Health Organisation [2] reports that only around 50% of the general population in developed countries are adherent to long-term therapies. Non-adherence can have a negative impact on the efficacy of treatments, patient well-being and the use of scarce healthcare resources [3,4]. The importance of finding ways to increase adherence is highlighted by Haynes and colleagues [5] in a systematic review of adherence interventions: “*Increasing the effectiveness of adherence interventions may have a far greater impact on the health of the population than any improvement in specific medical treatments*”.

However, this is not simply a matter of patients choosing not to take medicines as prescribed (intentional non-adherence) or experiencing difficulty with taking medicines (non-intentional non-adherence), since there are recognised to be a wide variety of factors that shape the landscape within which patients take medicines [6-8]. These range from factors that are concerned with interactions with health professionals to those that are related to broader societal issues [6]. The interplay between these factors is highly complex and no single intervention or approach has been shown to adequately address all of these issues [6]. Over at least the last three decades a vast but often contradictory body of literature on medicines adherence has accumulated that bears testimony to this [1,9]. As such, numerous gaps in knowledge remain and clear research-based evidence of how to reduce non-adherence on a large scale remains elusive. This suggests that a comprehensive and integrated approach is required to developing an evidence-informed strategy, which includes action at health policy level within and across nations.

One approach that is recognised to be of value in dealing with complex issues like this, where there are known to be numerous highly complex and inter-related factors involved and where uncertainties inevitably remain, is to harness expert opinion through consensus building [10-13]. The Delphi method [10] is used to ascertain expert opinion and build consensus through a series of ‘rounds’ of structured questioning with feedback at each stage. The technique enables a wide range of expertise on a particular issue to be collated and is ideally suited to electronic group communication when participants are widely geographically dispersed [14]. Participants retain anonymity throughout the Delphi process to minimise the influence of identity in their responses. This Delphi study, conducted as part of the European Commission funded Ascertaining Barriers to Compliance Project (ABC Project), was used to amass expert opinion on the causes, consequences, and solutions to medication non-adherence across Europe and develop consensus on the relative importance, and operational and political feasibility of potential solutions. In the absence of high-quality, consistent research evidence regarding the efficacy of adherence interventions, consensus evidence is a useful and appropriate resource for policy formulation.

## Methods

### Study design

The Delphi study proceeded over a series of four rounds held between January and June 2011. All rounds were completed online using Survey Monkey software and participants received feedback electronically via blind carbon copied emails. A consensus meeting with ABC project partners and members of the Delphi Panel took place at the end of the study to further develop the policy solutions. Conduct of the study was compliant with the Helsinki Declaration and the study received ethical approval from Keele University Ethical Review Panel.

### The Delphi expert panel

Purposive sampling ensured that potential participants had expertise relevant to one or more of the five dimensions of adherence in the World Health Organisation model [2]. Panel members were sampled from the following five stakeholder groups to ensure a broad range of expertise: academic; healthcare commissioner or policymaker; pharmaceutical industry representative; patient, carer, or patient organisation; healthcare professional. European Medicines Agency representatives were also invited to take part. Participants were nominated by ABC Project researchers or identified through an extensive internet-based search. 50 individuals from 14 countries participated in one or more rounds of the study. There were 25 males and 25 females on the Panel. All five stakeholder groups were represented on the Panel and several Panellists belonged to more than one group. The European Medicines Agency was also represented.

### The Delphi rounds

#### Round 1

Three open-ended questions were presented to the Panel: What do you think the reasons are why people do not take their medicines as prescribed?; What do you think the consequences are of medicines non-adherence?; What do you think the solutions are to medicines non-adherence? Participants were asked to write as much or as little as they wished in response to each question.

For each of the three questions posed to the Panel in Round 1, the researchers independently segmented each panellist’s responses into a series of discrete statements. Statements were then coded and assigned to emerging categories in a series of refinements of the categorisation. Statements that were identical or very similar in essence were collapsed to form a single statement. Validation of the categorisation process involved discussing the features of each category to ensure distinctiveness, establishing agreement that category names reflected the statements that they subsumed, and scrutinising statements that were not initially placed in a category for congruence with existing categories. Some statements were not categorised including general introductory text around the subject, humorous remarks, and direct repetition of an earlier statement within the participant’s response. The researchers remained blind to the authorship of statements throughout.

#### Round 2

All panellists received feedback on the Panel’s responses to the three questions from Round 1 and instructions for the next round. Round 2 focused on the Panel’s proposed solutions to non-adherence from the first round by inviting participants to agree, reject, or amend each proposed solution and offer new solutions that had not been advanced in Round 1.

An *a priori* criterion for refining the solutions specified that those rejected by more than 50 per cent of the Panel should not be taken forward to Round 3. Where amendments were proposed, data from Round 1 were revisited to seek examples, clarification, or support for suggested modifications that could be made without changing the core meaning of statements.

#### Round 3

Participants received feedback about the Panel’s collective responses to Round 2 and were asked to rate the importance, and operational and political feasibility of each solution. Ratings were made on five-point Likert-type scales, as shown in TableÂ 1.

**Table 1:** **Response scales used by the Delphi Panel for rating the importance**, **operational feasibility, and political feasibility of solutions to medication non-adherence**

**Important scale**	**Operational feasibility scale**	**Political feasibility scale**
**1. Not at all important**	**1. Definitely unfeasible**	**1. Definitely politically unfeasible**
− Unlikely to have impact on non-adherence	− Cannot be implemented	− Politically unacceptable
− Not at all confident about effectiveness of solution	− Unprecedented allocation of resources would be needed	− Completely unacceptable to the public
− Basic research needed		
**2. Slightly important**	**2. Probably unfeasible**	**2. Probably politically unfeasible**
− Potential for impact on a minority of patients	− Some indication that this cannot be implemented	− Major political obstacles
− Not very confident about effectiveness of solution	− Large scale increase in available resources would be needed	− Not acceptable to a large proportion of the general public
− Major research effort needed		
**3. Somewhat important**	**3. May or may not be implemented**	**3. May or may not be implemented politically**
− Potential for impact on some patients	− Contradictory evidence that this can be implemented	− Political obstacles
− Unsure about effectiveness of solution	− Increase in available resources would be needed	− Some indication that this may not be acceptable to a large proportion of the general public
− Indeterminable research evidence available		
**4. Very important**	**4. Probably feasible**	**4. Probably politically feasible**
− Potential for impact on majority of patients	− Some indication that this could be implemented	− Some minor political obstacles
− Quite confident about effectiveness of solution	− Available resources would have to be supplemented	− Further consideration may have to be given to public reaction, although some evidence exists that the proposed solution may be acceptable
− Some research still required		
**5. Extremely important**	**5. Definitely feasible**	**5. Definitely politically feasible**
− Potential for widespread general impact	− Can be implemented	− No major political obstacles
− Very confident about effectiveness of solution	− Necessary resources (financial, labour etc) are presently available	− Will be acceptable to the general public
− No further research required		

The importance rating scale was adapted from Hardy et al. [15], while the feasibility scales were adapted from Adler and Ziglio [10].

There are no definitive guidelines for establishing consensus in Delphi literature. While some researchers have suggested that 51% agreement among respondents can be interpreted as indicating consensus [16,17], others have adopted more stringent levels [18]. Prior to data collection [11,19], we defined consensus as 75 per cent or more responses falling within a two-point bracket on a response scale [15,20]. For instance, if 75 per cent or more respondents provided ratings of “3” (somewhat important) or “4” (very important) on the importance scale for a particular solution, this was deemed to represent consensus of the Panel on the importance of that solution.

#### Round 4

The purpose of this round was to seek consensus for ratings on which the Panel had not converged in Round 3, in light of information about panellists previous ratings. Panellists received the potential solutions to non-adherence for which consensus had not previously been reached, alongside the mean ratings of the Panel from the previous round. Participants who had taken part in Round 3 were also provided with a reminder of their own previous ratings for the potential solutions. Participants were asked to re-rate those particular dimensions for which consensus had not been achieved. All participants were invited to comment on their Round 4 ratings.

Means and standard deviations were calculated for all ratings made across Rounds 3 and 4. To produce a set of solutions to non-adherence that reflected the priorities of the Panel, only those solutions considered to be “very important” or “extremely important”, i.e., those with an importance rating of 4.00 or higher, were taken forward to the consensus meeting. Overall priority ratings for these policy solutions were calculated by summing the mean importance score and the average of the two mean feasibility scores for each solution.

### The consensus meeting

A group of 40 Delphi panellists and ABC Project researchers from 10 countries met at the Royal Society, London in June 2011. All stakeholder groups from the Delphi study were represented. The objective of this meeting was to further develop the policy solutions through group discussion. A plenary session at the end of the meeting enabled the chairs of each discussion group to present key outcomes of the roundtable discussions. The discussions were audio-recorded with participants’ consent.

The recordings of the plenary sessions were transcribed verbatim and analysed by two researchers who had not participated in the discussions. Key themes, ideas, and recommendations for policy development were extracted. The policy solutions were amended in light of the recommendations for development that had been agreed within participants’ discussions.

## Results

### Round 1

The three questions generated a total of 1,142 statements; 531 statements in response to question 1, 256 for question 2, and 355 statements in response to question 3, from which 501, 244 and 343 statements, respectively, were extracted for analysis. Summaries of responses by category for each question are shown in TablesÂ 2, 3, and 4.

**Table 2:** Causes of medication non-adherence by category

**Category**	**Count**	**%**
Patient factors - patient behaviour/characteristics	100	20.0
Medication factors	98	19.6
Patient factors - treatment effects	66	13.2
Patient factors - patient beliefs and concerns	55	10.9
Clinician factors	42	8.4
Meta theories of adherence/ theories of adherence	42	8.4
Healthcare organisation factors	32	6.4
Patient/clinician interaction	29	5.8
Environmental and social/structural factors	28	5.6
Disease factors	9	1.8
Total	501	

**Table 3:** Consequences of medication non-adherence by category

**Categories**	**Number**	**%**
Themes/theories	20	8.2
Consequences for patients	125	51.2
*Disease consequences*	*54*	
*Medication consequences*	*46*	
*Quality of life/well-being consequences*	*25*	
Consequences for healthcare professionals	6	2.5
Consequences for clinician-patient interaction	11	4.5
Consequences for the healthcare system	62	25.4
*Waste of resources*	*52*	
*Public health risk*	*10*	
Consequences for society	20	8.2
*Waste of resources*	*14*	
*Research/industry*	*6*	
Total	244	

**Table 4:** Solutions to medication non-adherence by category

**Solution Category**	**Number**	**%**
Patient focused solutions	187	54.5
*Educational/informational*	*107*	
*Medication-related*	*39*	
*Behavioural strategies to eradicate forgetfulness etc.*	*32*	
*Involvement of the social network/caregivers can supportpatients with medication adherence*	*6*	
*Building the patient’s trust in the healthcare professional would improve medication adherence*	*3*	
Healthcare professional focused solutions	69	20.1
Clinician-patient interaction focused solutions	43	12.5
Themes/theories relating to solutions	16	4.7
Health system solutions	14	4.1
Government focused solutions	14	4.1
Total	343	

#### Causes of non-adherence to medication

Approximately 43% of the causes of non-adherence concerned aspects of patients’ behaviour, beliefs or characteristics. These included forgetfulness, low health literacy, and negative beliefs about medicines. Patients’ experience and interpretation of treatment, such as perceptions of feeling no benefit from treatment and, conversely, stopping treatment when benefit was experienced, were also perceived as causes of non-adherence. Medication itself was a cause of non-adherence in nearly 20% of statements, because of the complexity of medication regimens, polypharmacy, the cost of medication for the patient, and side effects. Overarching theories about the causes of medicines non-adherence represented a significant minority of statements. These often referred to the multiplicity of factors that together can result in non-adherence, and the notion that medicines non-adherence can be intentional and unintentional.

#### Consequences of non-adherence to medication

Panellists viewed the consequences of medication non-adherence to be overwhelmingly, but not exclusively, negative. Over half of the consequences listed by the Panel were experienced directly by the patient, positive or negative, through symptom experience, disease progression, quality of life, illness and death. A quarter were experienced by the healthcare system, through waste of money, medication and resources and increased utilisation of healthcare.

Although the consequences of medication non-adherence were largely perceived as negative by the Panel, positive consequences of medication non-adherence were also listed. Positive consequences for patients included the avoidance of adverse/side effects resulting from medication use. Patient quality of life was also seen to benefit from medication non-adherence through feeling that one is not dependent on medication, and feelings of control and mastery.

#### Solutions to non-adherence to medication

More than half of the solutions offered by panellists focused on achieving change in patients’ knowledge and behaviour. There were nearly three times the number of statements relating to changing or adapting patients’ knowledge and behaviour than those relating to changing or adapting healthcare professionals’ education and behaviour. Solutions focusing on change at the healthcare system or government level together amounted to less than 10% of the solutions generated by the Panel.

A substantial proportion of statements by panel members emphasised the need to improve patient education and information about treatment to make the information understandable, impartial, evidence-based, and inclusive of details of other forms of treatment. A similar number of statements described improving education and information about the administration of medication. Several statements specified the need to improve education and information on the potential side effects of the medication, while others expressed the importance of improving education and information to inform patients’ risk-benefit analysis.

A sub-set of solutions related to changes to medication, including simplification of the regimen, the development of better drugs with reduced side-effects, and improved packaging, and often referred to the tailoring of dosage to individual need and to compatibility with the patient’s lifestyle.

Improving education and training for identifying and assessing medication non-adherence was a prominent solution for the Panel. A number of statements focused on improving education and training in patient-centred care to move away from a paternalistic approach to patients. A large number of statements about the input of healthcare professionals related to the provision of ongoing feedback and support with medication-taking. Frequently cited amongst the healthcare professional-focused solutions was the importance of taking a non-judgmental approach and ending the conception of non-adherence as something that should be blamed on patients. The final category of solutions pertaining to healthcare professionals was the use of reviews of medication and included suggestions such as targeting reviews towards patients on multiple medications or complex regimens.

Solutions concerning clinician-patient interaction were fewer in number than those relating to healthcare professionals. Ensuring patient involvement and a partnership approach between clinicians and patients, building a partnership between doctors and patients, and the provision of frequent opportunities for open discussion with the patient about medication-taking were frequent statements here. The need to discuss patients’ beliefs about medications, the condition, and the likelihood of taking medications was also expressed. Statements about discussing patient preferences formed another category.

A small number of solutions referred to the impact of the health system on adherence behaviour and these fell into four categories. The first was a team approach to treatment by healthcare professionals, for example, the involvement of nurses and pharmacists, and the concept of the ‘Medicines Education Team’. The second category concerned financial investment for supporting adherence. The last two categories within the scope of the healthcare system are in opposition; while the larger of the two contained statements about reducing the cost of medications for patients through the development of reimbursement systems, removing prescription charges, and lowering out of pocket costs for medication, the other encompassed statements about financial penalties for the non-adherent patient.

A few solutions related to government involvement in adherence support. Three categories emerged: statements relating to the investment of resources or money in medication adherence, particularly regarding education, research, and access to medicines; increasing public awareness of the issue of medication adherence, for instance through public education campaigns and interventions to improve health literacy; and suggestions for policy development in the field of adherence, for example, elevating patient adherence as a critical healthcare issue.

Three overarching themes on solutions to non-adherence emerged from the data. These were not solutions but rather ‘meta-theories’ about the nature of solutions to non-adherence. The most commonly cited theme related to the complex nature of solutions and the need for multifaceted interventions to achieve a comprehensive response to non-adherence. Several panellists also highlighted the lack of long-term effectiveness of current solutions to non-adherence, and the absence of an evidence base for the effectiveness of adherence interventions. The final theme was the need for solutions to correspond to reasons for non-adherence, for example, developing solutions matched to unintentional or intentional causes of non-adherence.

### Round 2

For each of the 43 proposed solutions presented in Round 2, the percentage of the Panel that agreed with, rejected, or amended the solution was calculated. Two solutions that did not meet our a priori criterion for inclusion were deleted (“A consistent size, shape, and colour for medications throughout Europe, whoever the manufacturer is, would improve medication adherence” and “Financial penalties imposed on patients who are non-adherent would address medication adherence”).

### Round 3

The Panel achieved consensus for 64 of the 126 ratings. One solution was removed following feedback from panellists: “Patients need to broadly adjust to their illness and treatment”. This solution represented a suggestion put forward by one member of the panel in the first round and was queried by a number of panellists in Round 2. The Delphi research team therefore modified the wording of this solution to increase its clarity in Round 3. However, as a consequence of the concerns raised by several members of the panel with regard to this recommendation, this item was excluded from Round 4.

### Round 4

Means and standard deviations for the 60 ratings that were made in Round 4 were calculated. The criterion used for determining consensus in Round 3 was used in Round 4 to determine consensus (75% of ratings falling within a two-point bracket on the response scale). A substantial shift towards convergence of ratings was found, with 58 of the 60 ratings achieving consensus. Overall, 121 of the 123 ratings made across Rounds 3 and 4 achieved consensus.

Prioritised solutions were identified and ranked in a two-step process. Only those solutions considered to be “very important” or “extremely important” by the Expert Panel (i.e., with a mean rating of 4.00 or higher) were retained. The application of this criterion resulted in 25 policy solutions remaining in the final list. In order to determine the level of priority of each of these 25 solutions, the importance and feasibility ratings were combined in a single score by summing the mean importance score and the average of the two mean feasibility scores for each solution, as shown in TableÂ 5.

**Table 5:** The ABC Delphi Panel medication adherence policy solutions

**Policy solutions**	Priority rating*	**Mean importance♦**	**Mean operational feasibility**	**Mean political feasibility**
1. Improve patient education and information when a medication is newly prescribed	8.92	4.47	4.39	4.50
2. Improve patient education and information focused on the patients’ treatment	8.42	4.13	4.16	4.42
3. Improve patient education and information regarding the benefits of adherence to their particular medication(s)	8.40	4.11	4.24	4.34
4. Improve education and training for healthcare professionals about ways of addressing medication non-adherence to drive improvements in clinical practice	8.32	4.42	3.86	3.93
5. The patients' preferences for treatment should be discussed to support medication adherence	8.27	4.32	3.89	4.00
6. Improve education and training for healthcare professionals about patient-centred care	8.25	4.32	3.89	3.96
7. Improve patient education and information about potential side effects or adverse effects and how to manage them	8.21	4.08	4.13	4.13
8. Healthcare professionals should support patients with concerns about or experience of side effects of medication	8.18	4.18	3.96	4.04
9. Improve education and training for healthcare professionals about identifying and assessing medication non-adherence to drive improvements in clinical practice	8.06	4.18	3.76	4.00
10. Ensure patient involvement and a partnership approach, for example in treatment plans and decisions, to support medication adherence for those patients who wish to be involved	8.05	4.32	3.66	3.79
11. Simplify the patients’ medication regimen (e.g., less frequent, modified formulation and/or dosage, tailored to individual need)	8.05	4.16	3.82	3.96
12. Improve education and training for healthcare professionals regarding medication adherence in general	8.03	4.05	3.95	4.00
13. Improve patient education and information to assist the patient to weigh up the benefit and harm of medication	7.99	4.18	3.75	3.86
14. Increase public awareness of the issue of medication adherence	7.94	4.13	3.82	3.79
15. The patients' health- and medication-related beliefs should be discussed between the clinician and the patient to support medication adherence	7.90	4.29	3.50	3.71
16. Healthcare professionals should use reviews of medication to discuss medication adherence with patients	7.84	4.03	3.82	3.79
17. Healthcare professionals should provide the patient with ongoing feedback and support with medication-taking	7.82	4.07	3.79	3.71
18. Stop medication(s) that the patient no longer needs or wants	7.81	4.00	3.75	3.86
19. Ensure a consistent team approach to treatment, in which all members of the healthcare team work together to support medication adherence	7.61	4.21	3.18	3.61
20. Healthcare professionals should adopt a non-judgmental approach to the issue of medication adherence	7.61	4.11	3.43	3.57
21. Build patients’ trust in the healthcare professional to support medication adherence	7.60	4.11	3.43	3.54
22. Information provision should be tailored to the individual preferences or needs of the patient	7.56	4.03	3.34	3.71
23. Governments should implement evidence-based policies about medication adherence	7.53	4.05	3.42	3.53
24. Governments should invest resources/money in medication adherence, particularly regarding education, research, and access to medicines	7.39	4.11	3.34	3.21
25. Healthcare professionals should make sufficient time for the patient, for instance through more frequent contact	6.79	4.00	2.76	2.82

* Higher ratings indicate higher priority; lowest possible priority rating = 2, highest possible priority rating = 10.

**♦** Importance, operational feasibility, and political feasibility ratings were made on 5-point scales; higher scores indicating higher importance/feasibility.

*Note.* All ratings for the policy solutions listed in the table achieved consensus from the ABC Delphi Panel.

### Consensus meeting

Minor amendments were made to the wording of policy solutions on the basis of themes identified from transcripts of final plenary statements at the consensus meeting and from discussion with the wider ABC project team. The resulting dissemination statement of consensus-based policy solutions for medication adherence is shown in TableÂ 6.

**Table 6:** ABC consensus-based policy solutions for medication adherence for Europe

	
**Patients benefit when provided with support, education, and information**	
**·**	when a medication is newly prescribed
**·**	focused on the patients’ treatment
**·**	about the benefits of adherence to their particular medication(s)
**·**	about potential side effects or adverse effects and how to manage them
**·**	to assist the patient to weigh up the benefit and harm of medication
**·**	tailored to the individual preferences or needs of the patient
**Healthcare professionals should receive education and training about**	
**·**	patient-centred care
**·**	identifying and assessing medication non-adherence
**·**	ways of addressing medication non-adherence when it is identified
**so that they can:**	
**·**	adopt a non-judgmental approach
**·**	identify medication non-adherence
**·**	provide patients with ongoing feedback and support with medication-taking
**·**	support patients with concerns about, or experience of, side effects of medication
**·**	make sufficient time for the patient, for instance through more frequent, timely contact
**Together, healthcare professionals and patients should**	
**·**	discuss the patients' preferences for treatment
**·**	ensure a partnership approach in decision making and treatment
**·**	discuss the patients' health- and medication-related beliefs
**·**	build the patients’ trust in the healthcare professional
**Regarding medicines**	
**·**	simplify the patients’ medication regimen as appropriate (e.g., less frequent, modified formulation and/or dosage, tailored to individual need)
**·**	stop medication(s) that the patient no longer needs or wants
**Healthcare providers should**	
**·**	promote a team approach, sharing information to deliver consistent adherence support
**·**	prioritise medication adherence support in service, organisation, and systems design
**Governments/healthcare payers should**	
**·**	increase public awareness of medication adherence for all citizens
**·**	develop and implement evidence-based interventions for medication adherence
**·**	provide training and guidance for all healthcare providers so they can deliver effective adherence interventions
**·**	invest in research to identify effective interventions demonstrating value for money

## Discussion

The Delphi panellists achieved consensus about a broad range of policy solutions for Europe to address medication non-adherence, and agreed on the relative importance and feasibility of those solutions. This consensus is all the more significant having been obtained with participants from fourteen countries from a diverse range of stakeholder groups who might be expected to have divergent perspectives, experiences and interests in medication adherence. Participation in a Delphi study demands a significant commitment of time and effort over a number of months and the 1,142 separate statements made by this Panel represent a significant resource.

### Strengths and limitations

Many, but not all, of the solutions target action at the patient and the public, rather than policy interventions at the healthcare provider or systems level. This may well correspond with the Panel’s beliefs, reflected in the wider research literature that the primary cause of non-adherence is patient’s beliefs about illness and treatment [21,22]. Equally, the majority of published adherence interventions are educational and behavioural interventions to change patient behaviour rather than (potentially more challenging) interventions to change healthcare systems and culture [23]. When considering potential solutions to non-adherence, panellists may have brought to mind those causes and interventions for non-adherence with which they are most familiar from research literature and practice, hence the focus on patient-oriented solutions over other ways of intervening to address medicines non-adherence.

For the purpose of this task, panellists were instructed to think about each potential solution, and in later rounds, the importance and feasibility of each solution, in isolation. In practice, interventions to support medicines adherence may be multi-faceted, delivered in parallel, and cut across the categories used here to help structure the task for participants. This study does not tell us how policy makers and commissioners might seek to combine interventions for best effect or perhaps stagger the introduction of individual solutions within an overall implementation strategy.

The requirement for panellists to communicate in the English language may have prevented some potential panellists from participation. Panellists came from 14 different countries, though a significant minority of the panel were UK based. Nevertheless, the policy solutions represent the combined views of a diverse group of stakeholders with a remit for adherence, rather than the views of a specific interest group or a single professional group [24,25].

This study improves on previous research initiatives to develop policy recommendations for medicines adherence in several ways. The proposed solutions look beyond the actions and interactions that occur in the clinical setting [7] to broader systems and process factors that impact on medicines adherence. Recommendations for policy to address medication adherence identified here for Europe are similar in scope to policy initiatives in the USA [26,27], which have also been developed with multi-stakeholder input.

### Comparison with other studies

Other research in this area, such as systematic reviews of adherence interventions [5], tends to report that the evidence for the effectiveness of adherence interventions is either limited, short lived in duration of effect, or both. Many adherence interventions in research studies are complex and it can be difficult to tease apart the active ingredients of the intervention. A challenge for healthcare policymakers is overcoming the stark gap between interventions that are delivered as part of clinical trials and the reality of what is possible in clinical practice and within limited budgets. Our study provides succour for the policy maker seeking effective solutions for medicines non-adherence that are also feasible at an operational and political level. On the latter point, the research evidence-base has little to offer. In this regard our study may act as a guide for evidence-informed implementation.

## Conclusions

The solutions to medication non-adherence for Europe are broad and practical in nature. The breadth of the policy solutions enables significant flexibility in national implementation to reflect differences in healthcare systems, health-related culture, available resources, and the level and sophistication of existing implementation. Local implementation of the highest priority item ‘improve patient and education when a medication is newly prescribed’ could, for example, be delivered in a number of ways: as part of a community pharmacy service such as the New Medicines Service in England [28]; in conjunction with a trial prescription programme such as those in Canada [29]; or within the context of existing services provided in primary care. The policy solutions described in TableÂ 6 have sufficient flexibility to incorporate a number of implementation responses. Policy recommendations for medication adherence for Europe have been discussed at several dissemination events as part of the ABC Project (further details at http://www.abcproject.eu/) and we hope this paper stimulates further discussion and action. Future efforts should focus on sharing implementation practice to improve our knowledge of the range of policy responses to medication non-adherence across Europe.

## Competing interests

The authors declare that they have no competing interests.

## Authors’ contributions

WC participated in the design of the study and was involved in data collection, data analysis, and data interpretation. SW was involved in the design of the study, data analysis, and data interpretation. SM participated in data collection, data analysis, and data interpretation. All authors helped to draft the manuscript and approved the final version.

## Authors’ information

All authors are based in the School of Pharmacy at Keele University. WC is Head of the Medicines Partnership Programme, SW is Lecturer in Pharmacy Practice, and SM is Medicines Partnership Research Assistant.

## Pre-publication history

The pre-publication history for this paper can be accessed here:

http://www.biomedcentral.com/1472-6963/12/425/prepub
